# Case Report: Endoscopic trans-cerebellar medullary fissure approach for the management of brainstem hemorrhage

**DOI:** 10.3389/fneur.2023.1173905

**Published:** 2023-07-06

**Authors:** Xue-Jian Wang, Longyao Zhang, Yu-Hua Yin, Zhi-Feng Wang, Yi Zhang, Cheng Sun, Zhi-Ming Cui

**Affiliations:** ^1^Department of Neurosurgery, Affiliated Hospital 2 of Nantong University, Nantong University, Nantong City, China; ^2^Department of Neurosurgery, Nantong Clinical Medical College, Kangda College, Nanjing Medical University, Nanjing, China; ^3^Department of Neurosurgery, Renji Hospital, Shanghai Jiao Tong University, Shanghai, China; ^4^Jiangsu Provincial Key Laboratory of Nerve Regeneration, Nantong University, Nantong, Jiangsu Province, China; ^5^Department of Orthopedics, Affiliated Hospital 2 to Nantong University, Nantong, Jiangsu Province, China

**Keywords:** endoscopic, trans-cerebellar medullary fissure approach to brain stem hemorrhage endoscopic, trans-cerebellar medullary fissure approach, brain stem, brain stem hemorrhage

## Abstract

**Objective:**

Brainstem hematoma (BSH) is a high-risk condition that can lead to deadly and disabling consequences if not properly managed. However, recent advances in endoscopic techniques, employed for removing supratentorial intracerebral hemorrhage have shown significant improvements in operative morbidity and mortality rates compared to other approaches. In this study, we demonstrate the utility and feasibility of the endoscopic trans-cerebellar medullary fissure approach for the management of brain stem hemorrhage in carefully selected patients.

**Patients and methods:**

A 55-year-old man presented to the emergency department in a comatose state with respiratory distress. A CT scan revealed the presence of a brainstem hemorrhage. Given the location of the hemorrhage and the need to quickly manage the associated developmental obstructive hydrocephalus and respiratory distress, an endoscopic trans-cerebellar medullary fissure approach was chosen as the most appropriate method of treatment.

**Results:**

Total resection was achieved, and the patient gradually improved postoperatively with no new neurological deficits. He is currently under routine follow-up and is conscious but has partial hemiplegia.

**Conclusion:**

This approach provided direct visualization of the lesion and was minimally invasive. The endoscopic trans-cerebellar medullary fissure approach may be considered an alternative to open approaches for brainstem hemorrhage in carefully selected patients.

## Introduction

Brain stem hemorrhage is a neurological condition that is characterized by a sudden onset, rapid disease progression, high surgical risk, high mortality, and disability rates, and a poor prognosis (1–3). Moreover, the mortality rate associated with brain stem hemorrhage is as high as over 50%, reaching as high as 100% in patients with hemorrhage volumes greater than 10 mL. Of all brain hemorrhages, pons hemorrhage is the most common type, accounting for approximately 90% of the cases. A large hematoma can cause a rupture of the fourth ventricle. In recent years, there has been significant progress in the fields of minimally invasive neurosurgery and neuroendoscopy. Reducing surgical trauma and improving surgical efficacy have become future goals for the development of a surgical treatment option for cerebral hemorrhage (2, 4, 5).

In the past, surgical intervention for brain stem hemorrhage was typically limited to cases with obstructive hydrocephalus. The brainstem is highly delicate, with a narrow surgical space. The location of the lesion requires that the surgical approach prioritize a safe surgical corridor and complete removal of the hematoma while minimizing manipulation of the neural tissue and vasculature (6, 7). The compact fiber tractography of the brainstem makes the surgical procedure more complex and limits the corridors available to the surgeon. However, with this knowledge, surgeons can use minimally invasive techniques to approach the lesion directly with minimal intraoperative trauma (2, 5).

More recently, endoscopic techniques have been employed to remove intracranial hemorrhage, significantly improving operative morbidity and mortality rates compared with other approaches (7). However, the removal of brainstem hemorrhage using endoscopic techniques has not been described yet. In this report, we present our experience with an endoscopic trans-cerebellar medullary fissure approach for the management of brainstem hemorrhage and a review of the relevant literature.

## Patients and methods

### Ethical approval and consent to participate

This research was approved by the Ethics Committee of our department. Informed consent was obtained, and this investigation was conducted in accordance with the principles of the Declaration of Helsinki. In addition, the authors obtained written informed consent from the patient.

**Figure 1:**
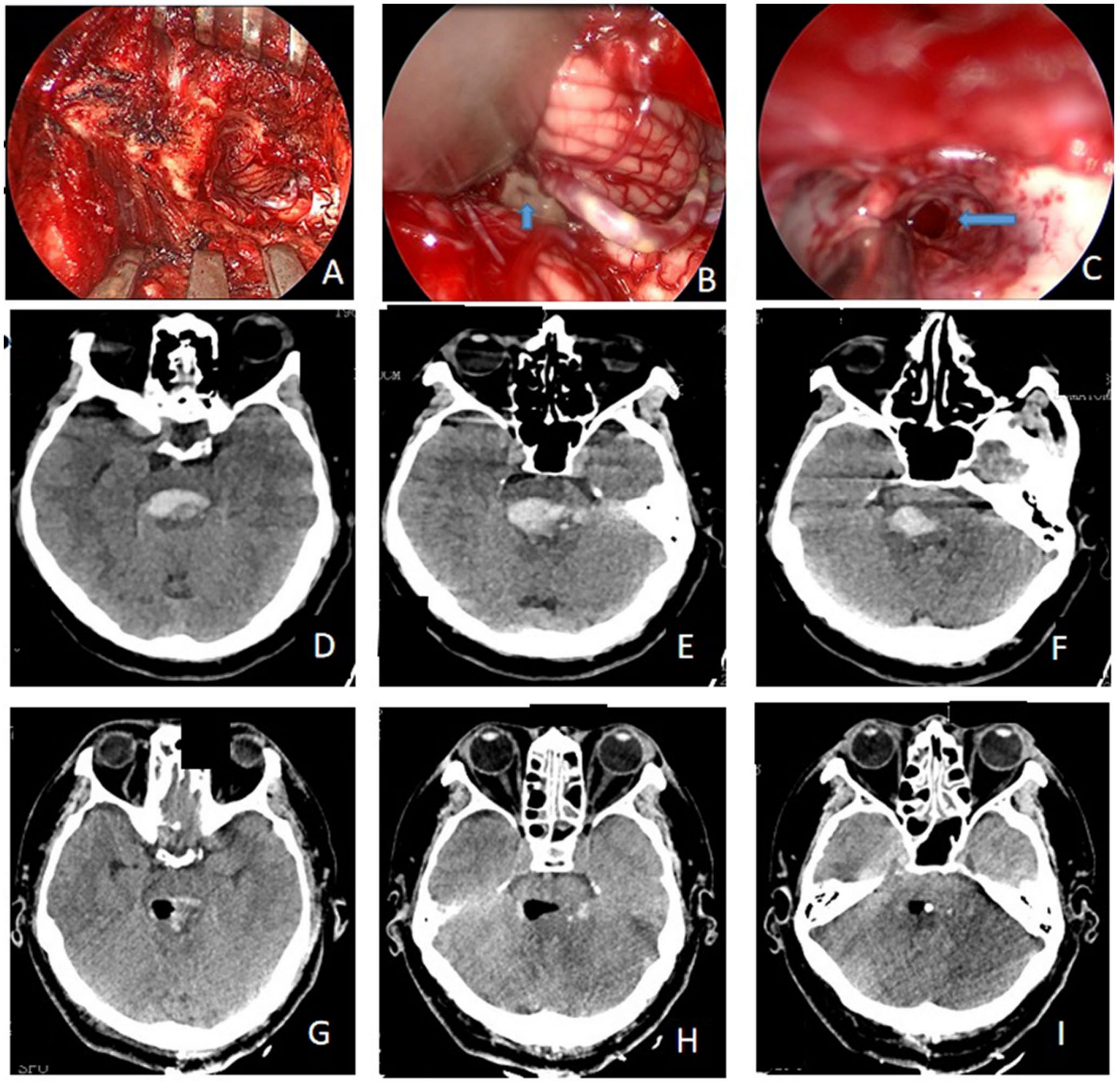
**(A)** Intraoperative incision and bone window; **(B)** the location of the hematoma was found during surgery (blue arrow); **(C)** the hematoma cavity after the removal of the hematoma by neuroendoscopy (blue arrow); **(D–F)** preoperative CT showed evidence of brainstem hemorrhage; **(G–I)** complete removal of hemorrhage was achieved, as confirmed by the post-op CT scan image.

On 4 October 2021, a 55-year-old man presented to the emergency department in a comatose state with a pinpoint pupil and respiratory distress. He had a history of hypertension for more than a decade. A CT scan revealed the presence of a brainstem hemorrhage (Figures 1D–F). Given the location of the hemorrhage and the need for timely management of associated conditions such as obstructive hydrocephalus and respiratory distress, the surgical team decided to use an endoscopic trans-cerebellar medullary fissure approach for intervention (Figures 1A–C).

**Figure 2:**
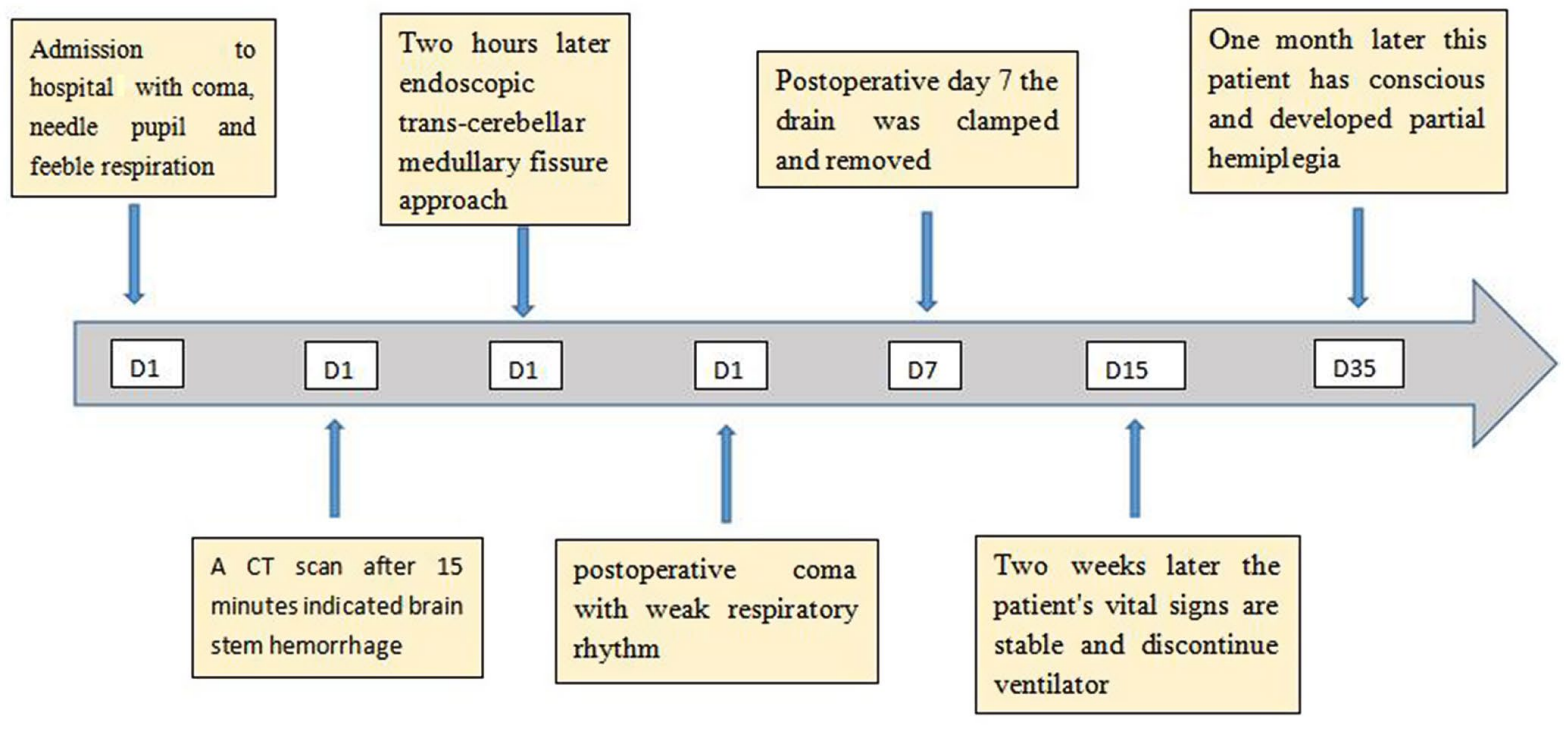
Timeline of the patient’s disease progression. The timeline illustrates the different events during the patient’s treatment and disease progression.

After 2 h of preparation, the patient was scheduled for a posterior median craniotomy, and image-guided complete removal of the hemorrhage was achieved, as confirmed by a post-op CT (Figures 1G–I, 2).

### Operative technique

After the induction of general endotracheal anesthesia, the patient was placed in a lateral prone position with his head secured in a 3-point Mayfield fixation. The patient’s head was slightly flexed, and his shoulders were cut open to provide the surgeon with direct access to the pillow neck.

The patient was treated with the suboccipital posterior median approach, which involved removing approximately 1.5 cm of the posterior margin of the foramen magnum. Using an endoscope, the cisterna magna was opened, and the cerebrospinal fluid was drained. The cerebellar tonsils were retracted to expose the lower part of the fourth ventricle, where the dorsal hematoma in the brainstem was located. A longitudinal incision was made to expose the hematoma cavity, and a suction device was used to gently and slowly remove the brainstem hematoma. Surgflo (Ethicon Inc., Somerville, NJ, USA) and Surgicel (Johnson & Johnson, USA) were used to prevent further bleeding, and the affected tissue was repeatedly washed with normal saline. The posterior fossa window was used to provide adequate decompression. Finally, the surgical team performed a puncture of the right occipital and lateral drainage ventricles to prevent supratentorial hydrocephalus.

## Results

The patient had a weak post-operative respiratory rhythm, which necessitated assisted ventilation with a tube ventilator. An immediate post-op CT revealed the trans-cerebellar medullary fissure surgical corridor and expected postoperative changes with complete removal of the hematoma and no evidence of acute hematoma or infarct (Figures 1D–F). The patient underwent right ventricular drainage at 150 mL/day for 7 days postoperatively. On the evening of the 7th day (11 October 2021), the drain was clamped postoperatively. After the CT scan confirmed that there was no evidence of hydrocephalus, the ventricular drain was removed. Two weeks later (26 October 2021), the patient gradually improved with no new neurological deficits, and the ventilator was discontinued. He then underwent rehabilitation training to improve his consciousness and was routinely monitored. After 1 month, he remained conscious but developed partial hemiplegia (28 November 2021).

## Discussion

Brain stem hemorrhage is characterized by a sudden onset, rapid disease progression, high surgical risk, high mortality and disability rates, and extremely poor prognosis. Brain stem hemorrhage is a challenging condition to manage due to its location near critical neural structures and poor surgical accessibility, resulting in a mass effect on surrounding neural tissues (6, 8). Historically, brain stem hemorrhage has been resected using a variety of approaches, including the transpeduncular, transpetrosal, or retrosigmoid approach (6, 9, 10). (Table 1) Irrespective of this, these routes are invasive and require a suboptimal working angle for the lesions.

**Table 1:** Simple comparison of the three methods of brainstem hemorrhage surgery.

Operation method	Puncture hematoma drainage	Microscopic craniotomy brain stem hematoma removal	Neuroendoscope craniotomy brain stem hematoma removal
Introduction	This method is puncture drainage of the hematoma cavity under the auxiliary measures	This type of surgery is performed under a microscope	This surgical approach is operated under neuroendoscopy
Advantage	Simple operation; Short time; Low blood loss; Trauma reduction; Small operation space; Less immediate bleeding	Direct view operation	Average complexity; modest operating space; Direct view operation
Disadvantage	Blindness; If there is active bleeding, it cannot be resolved	Complicated operation; Slow going; excessive bleeding; More traumatic; Large operation space is required	An average amount of time; Moderate bleeding; Medium trauma

In the past, conservative medical treatment was the preferred choice for managing brainstem hemorrhage, but it was not effective in patients with a severely large amount of bleeding or a broken fourth ventricle with the casting of the ventricle (2, 7). Brainstem hemorrhage not only directly destroys the life center of the brainstem but also stimulates the production of catabolic metabolites such as thrombin, which have toxic effects on neurons and nerve conduction bundles in the brainstem area. This can cause secondary damage to the brainstem and may aggravate cerebral edema, potentially leading to brainstem failure and a deterioration of the patient’s condition (11). Early removal of hematomas is crucial for preserving and restoring brain stem function. The goal of surgical treatment is to promptly eliminate the brainstem hematoma, reduce intracranial pressure, reduce pressure on the brain stem, and break the cycle of life-threatening bleeding.

In cases where acute obstructive hydrocephalus is caused by the casting of the fourth ventricle, external drainage of the lateral ventricle can help reduce intracranial pressure, but it cannot rapidly improve cerebrospinal fluid circulation (12, 13). Additionally, the compression caused by a brainstem hematoma cannot be alleviated quickly. Moreover, intracranial infections can be easily caused by an intraventricular canal and last for a long time. Stereotactic or neuronavigation-guided puncture and drainage can only remove part of the fluid bleeding, but cannot directly stop it, which may cause secondary brainstem trauma (2, 5). Ferroli et al. (14) argued that, if the lesion is located above the facial nerve nucleus plane, especially in the case of symptomatic brain stem hematoma, the surgical treatment effect is effective; if the hematoma is located on the ventral side of the brain stem or mainly in the medulla oblongata, the risk of surgery is significantly increased, and the postoperative effect is poor. In this case, the hematoma was located in the dorsal part of the brainstem and above the medulla oblongata, making surgery a viable option. The posterior operation provided significant advantages, and the prognosis was relatively good.

After the removal of the brainstem hematoma, catheter drainage was performed in the fourth ventricle. During surgery, when intracranial pressure was high, the surgeon removed approximately 1.5 cm of the posterior margin of the foramen magnum. This allowed for the opening of the cistern and the release of the bloody cerebrospinal fluid. The dura was then tensioned and repaired to allow complete posterior cranial decompression and prevent postoperative cerebral edema and possible cerebral infarction. The drainage tubes from the lateral and fourth ventricles were subsequently removed to prevent the occurrence of intracranial infection (15). No intracranial infection was observed in this patient after surgery.

Owing to its advantages of minimal invasiveness and direct visualization, neuroendoscopy has been widely used for managing cerebral hemorrhage (7, 16–18). In this particular case, neuroendoscopy was performed through the posterior median approach under the occipital bone. The advantages of neuroendoscopy, such as close monitoring, high-quality imaging, and wide observation area, are conducive to reducing intracranial injury, identifying brainstem rupture and internal bleeding points, avoiding secondary injury, reaching effective hemostasis, reducing the range of bone window, and also the pulling and injury of brain tissue (7, 19). In cases of high intracranial pressure, the posterior fossa of the occipital foramen was removed by biting, and the dura was sutured after reducing tension. The lateral ventricle and fourth ventricle external drainage were used to drain the bloody cerebrospinal fluid and open the CSF circulation, effectively reducing high cranial pressure. Postoperative, comprehensive treatment can significantly improve the prognosis of patients with brainstem hemorrhage.

## Conclusion

In selected cases, the endoscopic trans-cerebellar medullary fissure approach can be a highly effective method for treating brainstem hemorrhage. Compared to other techniques, it offers several advantages, such as reduced incision range and injury, direct visualization of the lesion, minimal invasiveness, and minimal manipulation of the neural tissue. However, the main drawback of this procedure is that it has a steep learning curve and requires an experienced multidisciplinary team. Despite these limitations, the endoscopic trans-cerebellar medullary fissure approach may be considered an alternative to open techniques for brainstem hemorrhage in carefully selected patients.

## Data availability statement

The original contributions presented in the study are included in the article/supplementary material, further inquiries can be directed to the corresponding author.

## Ethics statement

The studies involving human participants were reviewed and approved by Ethics Committee of the Second Affiliated Hospital of Nantong University. The patients/participants provided their written informed consent to participate in this study. Written informed consent was obtained from the individual(s) for the publication of any potentially identifiable images or data included in this article.

## Author contributions

X-JW and Y-HY treated this patient. YZ and Z-FW collected the data. LZ analyzed the data. X-JW wrote the manuscript and CS and Z-MC revised and checked the article. All authors contributed to the article and approved the submitted version.

## Funding

This study was funded by the Traditional Chinese Medicine Science and Technology Project of Jiangsu Province (YB2015113); the Science and Technology Program of Nantong Health Committee (nos. MA2019003, MA2021017, MB2021026, and MB2021027); the Science and Technology Program of Nantong City (nos. Key003, MS12015016, and JCZ2022040); and Kangda College of Nanjing Medical University (nos. KD2021JYYJYB025, KD2022KYJJZD019, and KD2022KYJJZD022).

## Conflict of interest

The authors declare that the research was conducted in the absence of any commercial or financial relationships that could be construed as a potential conflict of interest.

## Publisher’s note

All claims expressed in this article are solely those of the authors and do not necessarily represent those of their affiliated organizations, or those of the publisher, the editors and the reviewers. Any product that may be evaluated in this article, or claim that may be made by its manufacturer, is not guaranteed or endorsed by the publisher.
